# Healthcare providers’ experiences in supporting community-living older adults to manage multiple chronic conditions: a qualitative study

**DOI:** 10.1186/s12877-019-1345-2

**Published:** 2019-11-19

**Authors:** Jenny Ploeg, Marie-Lee Yous, Kimberly Fraser, Sinéad Dufour, Lisa Garland Baird, Sharon Kaasalainen, Carrie McAiney, Maureen Markle-Reid

**Affiliations:** 1 School of Nursing, Aging, Community and Health Research Unit, Faculty of Health Sciences and Associate Member, Department of Health, Aging and Society, McMaster University, 1280 Main Street West, Hamilton, ON L8S 4K1 Canada; 2 Faculty of Nursing, University of Alberta, Faculty of Health Disciplines, Athabasca University, 1 University Drive, Athabasca, AB T9S 3A3 Canada; 3 School of Rehabilitation Science, Faculty of Health Sciences, McMaster University, 1400 Main Street West, IAHS –403, Hamilton, ON L8S 4K1 Canada; 4 Faculty of Nursing, University of Prince Edward Island, 550 University Avenue, C1A4P3, Charlottetown, PEI Canada; 5 Schlegel Research Chair in Dementia, School of Public Health and Health Systems, University of Waterloo, 200 University Avenue West, Waterloo, ON N2L 3G1 Canada; 6 Aging, Community and Health Research Unit, Department of Health Research Methods, Evidence, and Impact, Faculty of Health Sciences, McMaster University, 1280 Main Street West, Hamilton, ON L8S 4K1 Canada

**Keywords:** Healthcare providers, Older adults, Multiple chronic conditions, Community care, Primary care, Qualitative research

## Abstract

**Background:**

Living with multiple chronic conditions (MCC), the coexistence of two or more chronic conditions, is becoming more prevalent as the population ages. Primary care and home care providers play key roles in caring for older adults with MCC such as facilitating complex care decisions, shared decision-making, and access to community health and support services. While there is some research on the perceptions and experiences of these providers in caring for this population, much of this literature is focused specifically on family physicians. Little is known about the experiences of other primary care and home care providers from multiple disciplines who care for this vulnerable group. The purpose of this study was to explore the experiences of primary and home care healthcare providers in supporting the care of older adults with MCC living in the community, and identify ways of improving care delivery and outcomes for this group.

**Methods:**

The study used an interpretive descriptive design. A total of 42 healthcare providers from two provinces in Canada (Ontario and Alberta) participated in individual semi-structured, face-to-face 60-min interviews. Participants represented diverse disciplines from primary care and home care settings. Inductive thematic analysis was used for data analysis.

**Results:**

The experiences and recommendations of healthcare providers managing care for older adults with MCC were organized into six major themes: (1) managing complexity associated with MCC, (2) implementing person-centred care, (3), supporting caregivers, (4) using a team approach for holistic care delivery, (5) encountering challenges and rewards, and (6) recommending ways to address the challenges of the healthcare system. Healthcare providers identified the need for a more comprehensive, integrated system of care to improve the delivery of care and outcomes for older adults with MCC and their family caregivers.

**Conclusions:**

Study findings suggest that community-based healthcare providers are using many relevant and appropriate strategies to support older adults living with the complexity of MCC, such as implementing person-centred care, supporting caregivers, working collaboratively with other providers, and addressing social determinants of health. However, they also identified the need for a more comprehensive, integrated system of care.

## Background

Multiple chronic conditions (MCC), defined as having two or more chronic conditions at the same time [1], have become a growing concern as the population ages [1–3]. It has been estimated that approximately 62% of older Americans who are aged 65 years or older have MCC [4]. However, prevalence rates vary widely, depending on how MCCs are defined and what types of chronic conditions are included [5, 6]. MCC is associated with an increased risk of mortality, functional decline, disability, poor quality of life, and harmful medication-related events [2, 4, 6, 7]. Research has shown that the number of health services used (e.g., primary care, home care, and acute care) and the associated healthcare costs increase with each additional chronic condition among older community-dwelling persons [8–11]. Not only is there an increased burden in terms of resource use, but MCC is also associated with burden and complexity in relation to the healthcare recommendations that healthcare providers should follow [12].

Typically, older adults with MCC receive care from multiple healthcare providers across various care settings [13]. For older adults with MCC living in the community, these healthcare providers are mainly from primary care and home care settings and include a broad range of providers such as nurses, physicians, social workers, pharmacists, physiotherapists, and personal support workers (or healthcare aides). Older adults with MCC and their family and friend caregivers (hereafter referred to as caregivers) experience their care to be focused on single conditions and lacking a holistic focus on the client and family [14]. Further, they feel that there is a lack of attention paid to their psychological and social needs, and that they are seldom involved in decision making related to their care [15].

Primary care and home care providers play key roles in caring for older adults with MCC such as facilitating complex care decisions, shared decision-making, and access to community health and support services. There is some research on the perceptions and experiences of primary care physicians in caring for this population [16–20]. Key findings are that: (a) physicians focus on medical problems rather than functional or social issues [16]; (b) there is little alignment of care goals between patient-caregivers and physicians [17]; and (c) mental health issues are seen to complicate the management of MCC [18]. Physicians described challenges to caring for this group such as the complexities of multiple interacting chronic conditions and the inadequacy of guidelines and evidence-based approaches that are typically based on individual conditions. However, this literature is focused specifically on family physicians and does not address the experiences of other primary and home care providers [16–20].

There is far less literature on the perspectives and experiences of other primary care providers such as nurses and this literature generally combines the perspectives of both nurses and family physicians [21–23]. Findings from this literature indicate that physicians and nurses: (a) reported difficulty in managing patients with MCC with limited consultation time [21, 23]; (b) included limited consideration of the interactions between conditions [21]; and (c) encountered conflicts between their own and patient goals [23].

Finally, there is very limited literature on the perspectives and experiences of home care providers who care for older adults with MCC [24]. A qualitative study conducted in Sweden sought to describe how professionals working for homemaker services and municipal and hospital-based home care services experienced collaboration in caring for older adults with MCC [24]. The study included nurses, physicians, an occupational therapist and a care administrator. Findings indicate that experiences of interprofessional collaboration were influenced by trust. Trust made it easier to collaborate with other healthcare providers when there were common goals, mutual respect and recognition of the skill of each profession. This study did not include unregulated home care workers (i.e., personal support workers), who are the largest group of home care providers. There is a need to better understand how home care workers collaborate with each other and professionals in primary care settings to support older adults with MCC.

In summary, there are few studies that examine the perspectives and experiences of a diverse group of primary care and home care providers (such as nurses, social workers, physiotherapists, personal support workers and others) in caring for older adults with MCC. Understanding the experiences of these healthcare providers as they seek to support older adults to manage their MCC is important as it may lead to enhanced practice approaches, improved patient outcomes and reduced unnecessary use of healthcare services.

The purpose of this study is therefore to explore the experiences of a broad range of healthcare providers working in primary care and home care settings in supporting older adults living in the community to manage their MCC. This paper reports on findings from a larger qualitative study that examined the experiences of older adults, family caregivers, and healthcare providers in managing MCC [14, 15]. This paper reports specifically on the findings of interviews with healthcare providers and sought to answer the following questions: (1) what are the experiences of healthcare providers in supporting community-living older adults to manage MCC? and (2) what are the recommendations of healthcare providers to improve care for older adults with MCC living in the community?

## Methods

### Design

We used Thorne’s qualitative methodology, interpretive description (ID) [25]. ID addresses clinical questions using an inductive approach to describe a phenomenon and understand it from the perspective of those experiencing it. ID is an approach that seeks to apply the new understandings to positively impact clinical care [25]. ID was consistent with our intent to provide an in-depth understanding of the experiences of healthcare providers in supporting older adults with MCC and supported the inclusion of illustrative participant quotes describing their experiences [25]. Philosophical underpinnings of the ID design and this research are that: (a) reality is subjective, constructed, contextual and complex; and (b) the researcher and researched interact to co-produce new understandings of a phenomenon [25].

### Sample and setting

We used purposive sampling strategies including criterion and maximum variation sampling [26]. For criterion sampling, healthcare providers were included in the study if they provided care to community-dwelling older adults aged 65 years and older who had three or more chronic conditions, consisting of at least one of the following conditions: dementia, diabetes, or stroke. These three conditions were selected because vascular diseases contribute to 30% of all deaths worldwide and place high burden on the older adult, their family, and the healthcare system [27]. Maximum variation sampling was used to obtain healthcare providers who had diverse healthcare backgrounds (e.g., nurses, physicians, social workers, personal support workers, physiotherapists, pharmacist) and worked in different community settings (e.g., primary care, home care). We included healthcare providers from two Canadian provinces; Alberta and Ontario. Both provinces have experienced a rapid growth in the proportion of older adults in their populations [28, 29].

### Recruitment

We recruited a broad range of healthcare providers from primary care and home care settings through partner sites in Alberta and Ontario. Designated individuals from partner sites were responsible for sending email invitations to their staff members or hard copy invitations if email was not available. Interested healthcare providers were asked to contact the research coordinator by phone or email to obtain more information about the study. The research coordinator contacted interested individuals to share study information and confirm eligibility. All participants received a $25 honorarium for participating in the study.

### Data collection

Data were collected using semi-structured, in-person one-on-one interviews from July 2013 to June 2014. Interviews were conducted by a research coordinator or research assistant who received training in conducting qualitative interviews and had experience in conducting qualitative interviews with healthcare providers. All interviews were audiotaped, and their average length was 60 min. Interviews took place at a time and place that was convenient for participants. A demographic questionnaire was used to collect information about participants such as age, gender, and professional background. An interview guide was developed based on a review of the literature and the expertise of the research team members (See Table 1). Data collection ended when we had some confidence that the complexity and variation of participant responses were addressing the research questions, acknowledging that there is always more to study on the topic [25].

**Table 1:** Interview Guide for Healthcare Providers

Experiences in Supporting Older Adults to Manage Multiple Chronic Conditions	
1. Tell me about your experiences in delivering care for older adults who have three or more chronic conditions.	
2. How do you help them manage multiple chronic conditions?	
3. How do you help older adults prevent their conditions from worsening or developing?	
4. How do you make decisions about managing multiple medications?	
5. What are the most important needs of family caregivers of older adults with multiple chronic conditions?	
6. How do you support family members of older adults with multiple chronic conditions?	
Facilitators in Managing Multiple Chronic Conditions	
7. What resources help you to manage multiple chronic conditions among older adults (e.g., people, community services, and financial resources)?	
Challenges in Managing Multiple Chronic Conditions	
8. What is your greatest challenge in supporting older adults to manage multiple chronic conditions?	
9. What makes it difficult to deliver care for them?	
Health and Social Services	
10. Do you refer older adults with multiple chronic conditions to any health and social services? If so, what are the services?	
11. What mechanisms are in place to enhance collaboration and coordination of care among multiple providers, services, settings, and sectors?	
Treatment Decisions	
12. How do you make decisions about managing more than one chronic condition at a time?	
13. How do you involve older adults and their family caregivers in making treatment decisions?	
Goals of Care	
14. What are your goals when caring for older adults with multiple chronic conditions?	
15. How do you develop these goals?	
16. What do you consider when developing these goals?	

### Data analysis

Digital recordings of interviews were transcribed verbatim by a trained transcriptionist. Transcripts were cleaned for accuracy by an experienced research assistant. Consistent with the ID design, we used the 6 steps of inductive thematic analysis as an analytic approach [30]. In becoming familiar with the data (Step 1), two research team members with qualitative expertise read through all transcripts and made notes of possible themes. In performing coding (Step 2), the two team members developed a coding scheme inductively derived from the data and met to reach agreement on the final coding scheme. One team member coded all transcripts using NVivo V.11.0 [31] to assist with data management. In seeking themes and reviewing themes (Steps 3 and 4), the two team members met monthly over three months to identify recurring and converging themes. Constant comparative analysis was used to identify similarities and differences in themes across participants, provinces, and settings (i.e., primary care, home care). The entire research team reviewed the themes and data within each theme and made suggestions for the final themes. We then created definitions of themes and named each theme (Step 5). Finally, we developed a written report of the final themes (Step 6).

### Methodological rigour

Credibility, described as the accurate reflection of participant experiences, [32] was achieved through: (a) including participants with diverse roles from two provinces in the identification of themes; and (b) investigator triangulation where analysis was conducted by investigators with expertise in qualitative approaches, older adults, multiple chronic conditions and community care. Transferability, described as the ability to apply findings to similar contexts, was addressed through a clear description of the participants, settings and research process [33]. Dependability was maintained as researchers kept field notes and a record of all analytic decisions. Confirmability to ensure that experiences remained grounded in actual events [32] was achieved by using direct quotes of participants to support study findings.

### Ethics

Ethics approval for this study was provided by the Hamilton Integrated Research Ethics Board (#13–411) in Hamilton, Ontario, Canada and the University of Alberta, Health Research Ethics Board (#39559) in Edmonton, Alberta, Canada, and renewed yearly as required. The procedures used in this study complied with the ethical standards of the Tri-Council Policy Statement, Ethical Conduct for Research Involving Humans [34]. Written informed consent was obtained from all participants by either a research coordinator or research assistant. Each participant was provided with a signed copy of the consent form.

## Results

### Demographic characteristics

A total of 42 healthcare providers participated in this study from Ontario (*n* = 22) and Alberta (*n* = 20) (See Table 2). Most participants were female (95.2%) and between the ages of 45 to 64 (59.5%). Participants represented a broad range of community healthcare providers including Registered Nurses, Registered Practical Nurses, Nurse Practitioner and Nurse Case Manager (50.0%), personal support workers (14.3%), physicians (9.5%), social workers (9.5%), physiotherapists (7.1%) and others. Most participants had more than 20 years of experience (40.5%) in their current working role. The percent of participants who worked in primary care (47.6%) and home care settings (45.2%) was similar.

**Table 2:** Healthcare Providers (*N* = 42)

Category	*n* (%)
Sex	
Female	40 (95.2)
Male	2 (4.8)
Age (years)	
18–44	17 (40.5)
45–64	25 (59.5)
Province	
Ontario	22 (52.4)
Alberta	20 (47.6)
Highest Education Completed	
Secondary School	1 (2.4)
Diploma	7 (16.7)
Bachelor’s Degree	17 (40.5)
Master’s Degree	7 (16.7)
MD	4 (9.5)
Certificate	6 (14.3)
Professional Background	
Exercise Therapist	1 (2.4)
Nurse Case Manager	1 (2.4)
Nurse Practitioner	1 (2.4)
Personal Support Worker	6 (14.3)
Pharmacist	1 (2.4)
Physician	4 (9.5)
Physiotherapist	3 (7.1)
Registered Dietitian	1 (2.4)
Registered Nurse	12 (28.6)
Registered/Licensed Practical Nurse	7 (16.7)
Speech Language Pathologist	1 (2.4)
Social Worker	4 (9.5)
Years of Experience in Current Role	
0–5 years	7 (16.7)
6–10 years	7 (16.7)
11–15 years	8 (19.0)
16–20 years	3 (7.1)
20+ years	17 (40.5)
Work Setting	
Primary Care	20 (47.6)
Home Care	19 (45.2)
Other (e.g., rehabilitation service, outreach clinic)	3 (7.1)

### Themes

Healthcare providers’ experiences of supporting older adults to manage MCC were characterized by six themes: (1) managing the complexity associated with MCC, (2) implementing person-centred care, (3), involving and supporting family caregivers, (4) using a team approach for holistic care delivery, (5) encountering challenges and rewards, and (6) recommending ways to address the challenges of the healthcare system (See Table 3). We found no differences in themes across provinces but did see some minor variation by setting (i.e., providers working in primary care and home care) and this is described in the sections below where applicable. Quotes are labelled with the profession of participants and ID number.

**Table 3:** Themes and Sub-Themes of Healthcare Providers’ Experiences

Themes	Sub-Themes
1. 1. 1. Managing the Complexity Associated with MCC	• Optimizing medication use: *“simplify their dosing regimens”*
	• Being proactive to promote health and prevent disease*: “minimize the risk factors with lifestyle”*
	• Recognizing and addressing the interrelatedness of health and social conditions*: “reducing the physical and social barriers”*
2. 1. Implementing Person-Centred Care	• Individualizing care: *“[care] catered to their individual needs”*
	• Enhancing quality of life: *“number one goal is to improve their quality of life”*
3. 1. Supporting Caregivers	• Educating caregivers to support older adults with MCC: *“it’s about education”*
	• Providing support and services for caregivers: *“linking caregivers to resources”*
4. 1. Using a Team Approach for Holistic Care Delivery	• Collaborating with multiple disciplines to provide holistic care: *“interdisciplinary collaboration is helpful and essential”*
	• Encountering poor team communication: *“information is not transferred in a timely fashion”*
5. 1. Encountering Challenges and Rewards	• Facing challenges in caring: *“the complexity in itself is more time consuming”*
	• Reaping the rewards of caring: *“seeing them stabilize or improve is always rewarding”*
6. 1. 1. Recommending Ways to Address the Challenges of the Healthcare System	• Improving care coordination: *“a more streamlined healthcare system”*
	• Improving primary care: *“longer more regular visits”*
	• Increasing home care supports: *“they need more home care”*

#### Managing the complexity associated with MCC

Healthcare providers described how they managed the complexity associated with supporting older adults with MCC. They sought to optimize the way medications were used to address the multiple and interrelated health conditions of older adults with MCC. They used proactive approaches to prevent conditions from worsening or new ones from developing such as promoting lifestyle changes and self-management, and supporting people to connect with health and social services in the community. They also recognized and addressed the interrelatedness of the older person’s health and social conditions.

***Optimizing medication use: “simplify their dosing regimens.”*** Healthcare providers sought to optimize the use of medications to address the complex healthcare needs of older adults with MCC. They frequently completed medication reviews to identify when older adults were or were not taking medications as well as opportunities for deprescribing in order to simplify often complex medication regimens: *“Reducing the amount of medications seniors are on and looking at polypharmacy …we’re always looking at their medication”* (Nurse Practitioner 9). Participants described their awareness of reasons why older adults did not take medications such as lack of understanding and forgetting to take medications.

*“…it becomes clear that compliance is an issue or there’s confusion about what they’re taking and they don’t understand, you know, what this medication is for, so they didn’t take it. I mean that happens a lot.”*

(Dietitian 6).

Healthcare providers such as physicians, pharmacists, and nurse practitioners felt that they often had to balance the need to treat chronic conditions with the potential risk of medication side effects for older adults.

*“I think it’s always a balance between over and under treating, certainly the frail elderly, we have to be careful about putting them on too many medications and then we actually cause more side effects that worsen their functional levels. But at the same time, you don’t want to under treat people who could benefit from medication.”*

(Physician 1).

Following multiple, disease-specific clinical guidelines in providing care was recognized as difficult as strict adherence to guidelines could lead to multiple, often interacting medications being prescribed.

Healthcare providers recognized the high costs of medications related to managing MCC. Some of their clients did not take their medications because they could not afford them. They acknowledged the out-of-pocket costs of medications and the fixed incomes of older adults and used strategies to address these issues.

*“We find out financing; we find out when they’re really taking their medications, which ones are being skipped; then we will work with our wonderful pharmacist to see which other meds that we can get them on that are paid and we can try and simplify their dosing regimens.”*

(Social Worker 30).

#### Being proactive to promote health and prevent disease: “minimize the risk factors with

***lifestyle.”*** Healthcare providers described proactive strategies they used to support older adults to manage MCC. For example, they focused on lifestyle behaviours to prevent the development of new, or worsening of existing, chronic conditions by promoting healthy eating, physical activity, smoking cessation, and social interaction. In doing so, they often addressed barriers to health promotion such as finances and transportation.

*“So things that we would do under the Chronic Disease Management Model may be refer them to an exercise specialist. Using the example of obesity, you say, “Can we refer you to an exercise specialist? Can we refer you to a dietitian?” And that way it’s not costing the patient any money; all they have to do is get themselves to and from the appointment. We find out, “Do you drive? If you don’t drive, here’s some resources that we have here.”*

(Nurse 12).

Healthcare providers described how they engaged older adults with MCC in proactively managing their care and equipped them with resources to achieve self-management goals: *“So the patients need to be committed and lot more emphasis is on self-management, encouraging the patients to participate and be more proactive themselves, rather than the patient coming to you and wanting you to fix them”* (Nurse Practitioner 9). Providers recognized that when older adults with MCC have a better understanding of their conditions they take more ownership in managing their conditions.

*“First of all, you want to ensure the person understands what their conditions are and so the more they understand it, hopefully the more ownership they’ll take over the condition and the better control they’ll have.”*

(Physician 11).

Participants also proactively referred older adults with MCC to other healthcare and community support services to help them manage MCC at home. Examples of support services included local Alzheimer societies, home care services, meal delivery services, and foot care services. Addressing social isolation was a proactive approach to preventing the worsening of conditions as it promoted better management of chronic conditions through peer support and motivation: “*I think just that socialization piece and the support that people can get from groups [group sessions] is really important in terms of managing chronic conditions as well”* (Social Worker 15).

***Recognizing and addressing the interrelatedness of health and social conditions: “reducing the physical and social barriers.”*** Healthcare providers recognized not only how MCC were interrelated, but also how these were interrelated with social, financial and other life circumstances. They acknowledged that health and social conditions changed over time and that they needed to address the complex relationship of conditions. Providers described conducting comprehensive and ongoing assessments to better understand the complexity of experiences of older adults. They considered the social situation of their clients and the level of involvement that caregivers have in helping to manage MCC.

*“I have a patient right now who was referred to me because of slight general mobility, she had pulled and hurt her ankle...and she has severe Alzheimer’s but she also has rheumatoid arthritis. So, just her husband...her spouse...and the caregiver stress that he has because there are certain things she can’t do and can’t manage because of the rheumatoid arthritis.”*

(Physiotherapist 8).

Providers were aware of the impact of social determinants of health in managing MCC and implemented strategies aimed at reduction of out-of-pocket costs for older adults with MCC and their family caregivers. They referred clients to community support programs that had little or no cost. They also provided older adults with options for affordable or free transportation to and from appointments or group sessions. Healthcare providers acknowledged that they could not address single conditions without considering the other conditions and circumstances facing the person as a whole. Healthcare providers helped support their clients in managing MCC by addressing social isolation and financial barriers.

*“But the honest truth is, to help their conditions from getting worse; it is usually, in our experience, helped by breaking their social isolation or by reducing the physical and social barriers to doing what they already know they should do. They already have very good information on what they should do but very often, they can’t do it. But they don’t want to tell the doctor that. “I can’t go out and buy this because I don’t have money for that,” or “I can’t go out and buy this because I don’t go out and buy anything anymore; it’s delivered.” So, it’s trying to reduce the physical and the barriers that they feel stigmatize them.”*

(Social Worker 30).

#### Implementing person-centred care

Healthcare providers explained that they implemented person-centred care when supporting older adults with MCC. They individualized care by recognizing and responding to the unique needs of older adults and considering their preferences for care. They promoted quality of life and supported functional abilities so older adults could better manage their own care.

***Individualizing care: “[care] catered to their individual needs.”*** Healthcare providers respected the preferences of older adults and their caregivers when making decisions about management of MCC. They engaged in discussions and prioritized goals of care with their clients and ensured that the care they provided addressed what was important to the person.

*“So, if somebody comes with multiple comorbidities…they’ve had a stroke, they have dementia, they have diabetes, there is an anxiety we’ll have to talk through it and say, “Okay, what’s at the front…what worries you the most?” And, we could get discussion from the patient, discussion from the family member and then determine what the issue is and what the goals are.”*

(Speech Language Pathologist 2).

When discussing options for managing care, providers ensured that older adults with MCC were aware of the benefits and risks of each choice so that they could make informed decisions. Providers also ensured that care was specific to the needs of older adults with MCC and that they were satisfied with the plan of care.

*“I offer them a bunch of options, whatever I can offer to them. I ask them to narrow it down and I give them my opinion which I think would be the best. And I’d say, “But the final decision is up to you, because if we offer you something that you’re not whole-heartedly wanting, then the chances of you continuing with that will be low, and we want to know that you [have] a care plan that’s going to work for you and you’re going to be happy with and you’re going to continue”*

(Nurse 25).

***Enhancing quality of life: “number one goal is to improve quality of life.”*** Healthcare providers described how they sought to enhance the quality of life of older adults with MCC and improve their physical and mental functioning. In making often complex treatment decisions, they recognized the need to prioritize quality of life over quantity of years lived.

*“I think optimizing their quality of life. Certainly we like to achieve the targets that our clinical practice guidelines suggest. But we always have to weigh that, you know, it’s quality over quantity in terms of years of life saved or what is the patient’s goal? It’s about what’s important to them.”*

(Nurse 4).

Providers focused on extending the independence and functional abilities of older adults with MCC. *“I always try, with every person, to have them maximize their function, whatever that function is”* (Physician 1). They supported client goals of aging at home whenever possible.

#### Supporting caregivers

Healthcare providers explained that caregivers were critical in supporting older adults with MCC to care for themselves and remain at home. They provided education to caregivers so they could better support the older adult in managing MCC. They also helped to link caregivers to community programs and services to support them in their caregiving roles.

***Educating caregivers to support older adults with MCC: “it’s about education.”*** Healthcare providers offered education to caregivers on how to best support the person with MCC at home through clinic or home visits, and group sessions. This education was often focused on improved understanding of disease processes, managing medications, and watching for signs and symptoms of worsening health of the older adult.

*“They [caregivers] need education and support. It’s very valuable for them to understand the medications and understand how the medications work for their family member. For example, patients can quickly turn [for the worse] and we can have more problems if the patient continues to take the medication if they’re ill.”*

(Nurse Practitioner 9).

Healthcare providers also involved caregivers in supporting older adults with self-care activities such as exercise: *“Oftentimes the caregiver might be a vehicle that we use to show the exercises and what they need to do and then obviously get them to support the patient to do them at home”* (Exercise Specialist 7).

***Providing support and services for caregivers: “linking caregivers to resources.”*** Healthcare providers recognized that caregivers of older adults with MCC often experienced stress and burden in their caregiving roles. They described offering emotional support for these caregivers. “*Information, guidance... give the family support. Let them know that they’re doing a great job”* (Personal Support Worker 17).

Healthcare providers also offered caregivers information about and referral to community resources to help them in caring for their family or friend with MCC. “*Linking [caregivers] to resources, to respite care, to make sure that they’ve got connections to those other outside supports that might be a benefit for them”* (Dietitian 6). They particularly focused on needs for respite and peer support.

*“So they need peer support but they also need respite. They need to be able to leave the person at home for seven or eight hours and go to their children or go and do whatever they want, so they need respite...not every day but for how often they want it, once every week, a four-hour block or twice a week.”*

(Social Worker 30).

Providers described how they used lists of community resources to share with caregivers and that they also considered services for the older adult (such as adult day programs) as sources of respite for caregivers.

*“We have a long list of resources in the community that we can provide for them; Meals on Wheels, transportation issues, voluntary visitors, all of those things that can make their life a little bit easier. If there was, like a direct need...if the caregiver agrees to have the client go to an adult day program once or twice a week that will give them time to do their own thing; give them a break...in this area, like yoga or exercise and that type of thing...support groups, caregiver support groups.”*

(Physiotherapist 8).

### Using a team approach for holistic care delivery

Healthcare providers indicated that they used an interdisciplinary team approach to holistically address the multiple and complex needs of older adults with MCC. While they noted the importance of regular and timely communication between providers, they encountered situations where poor communication occurred between team members. Healthcare providers discussed that information concerning older adults with MCC was not being transferred to other providers within the circle of care in a timely manner.

***Collaborating with multiple disciplines to provide holistic care: “interdisciplinary collaboration is helpful and essential.”*** Healthcare providers described how they used a collaborative team approach to provide holistic care for older adults with MCC. Almost all providers working in primary care discussed collaborating with multiple disciplines to provide comprehensive care, while only about half of the providers in home care discussed this. This suggests that there may be more barriers to collaboration among providers in home care, such as the largely independent nature of home visiting and communication challenges. Providers described the importance of integrating care from interdisciplinary team members to address the complex health and social care needs of older adults with MCC.

*“We have a good team kind of approach in that we all collaborate together and we come.*

*together all in a day to work together and come up with kind of care plans and meet with the.*

*family and the patient and the physician...and it truly is more of a team approach.”*

(Pharmacist 5)

Providers working in primary care teams valued having multidisciplinary team members on site to facilitate collaboration. “*Working as part of a team has been really great …doctors, pharmacist and now we have a kinesiologist who can help clients to be more active. I think it’s really important for managing a lot of chronic conditions*” (Social Worker 15). Providers also talked about working with community agencies, establishing collaborative working relationships so they could better support older adults with MCC: “*There’s a lot of formal and informal networking that happens. We often invite them to come to our team meetings to [understand] what new programs do you have*” (Nurse Practitioner 9).

***Encountering poor team communication: “information is not transferred in a timely fashion.”*** Healthcare providers described examples where the lack of a team approach hindered the care of older adults with MCC. This happened, for example, when specialists failed to communicate with family physicians in treating older adults (e.g., prescribing new medications) and when there was a lack of communication between healthcare providers. Home care providers indicated that they often experienced a delay in obtaining necessary health information about older adults with MCC prior to initiating home visits. Personal support workers indicated that they were often not included in team meetings yet they were the ones who provided the most care for clients at home and knew these clients better than other providers: “*I feel the PSW is not usually consulted in the meetings…and we’re the ones that other than the family members, we do have the most information*” (Personal Support Worker 17).

#### Encountering challenges and rewards

Healthcare providers described feeling challenged, drained and frustrated in caring for older adults with MCC. Despite these challenges, they also felt rewarded in being able to improve the lives of their clients and being appreciated by their clients.

***Facing challenges in caring: “the complexity in itself is more time consuming.”*** When caring for older adults with MCC, healthcare providers reported that “*their care needs can be overwhelming*” (Physician 1), and “*draining if they have a lot of conditions*” (Physiotherapist 8). They found it difficult to address the multiple and complex care needs of older adults with MCC in the limited time they had. Providers found it challenging to care for older adults with MCC as their conditions worsened and options for care became more limited. They also experienced ethical challenges in respecting the choices of clients when those choices led to negative health outcomes.

*“I think it’s allowing the patient to make bad choices...what we consider bad choices. Watching someone progress becomes a challenge. Watching someone losing their limbs one at a time becomes a challenge. When you try to do what you can but you know it’s ultimately the choices that people make; choices that we disagree with is really almost an ethical dilemma. So that is really hard. That would be our biggest [challenge].”* (Nurse 12).

Providers reported feeling frustrated when clients with MCC did not follow the care plans established by the primary care team.

Healthcare providers experienced challenges in supporting caregivers who were often stressed and overwhelmed with caring for older adults with MCC. Providers felt a strong sense of responsibility to address their needs but often suffered a heavy emotional toll from doing so.

*“Family relations...sometimes you get a lot of guilt, you know, coming from the caregiver as, you know, “I can’t do it anymore and I just feel awful but I can’t do it.” And they’re crying and it’s overwhelming and I have to sit and I have to console. You cannot walk away from that personal, emotional situation; no matter how late you are, you cannot walk out the door.”*

(Physiotherapist 27).

Healthcare providers reported challenges in accessing resources for older adults with MCC. They noted that providing care was difficult when financial resources were not available to support older adults with MCC in their own homes. Providers also described the lack of resources available to provide basic assistance such as accompanying clients outside the home.

*“I have been talking with the [agency] on and on and on...they have no volunteers here for that so I can’t get anyone to go into his home and wheel him out the front door and down the street once a week. I can’t get anyone to do that. So then I figured I would advertise that, so those kinds of things are very frustrating for us when it sounds like we have all these resources...and I know I’ve said we have tons...but some of them are bare.”*

(Social Worker 30).

***Reaping the rewards of caring: “seeing them stabilize or improve is always rewarding.”*** Healthcare providers experienced rewards when caring for this population, such as seeing improvements in the lives of older adults and feeling appreciated. All providers working in primary care and more than half of the home care providers discussed the rewards associated with caring for older adults with MCC. While living with MCC is often associated with deteriorating health, providers felt personally rewarded when their actions helped older adults improve their quality of life, improve functioning, and maintain an active and fulfilling social life.

*“I get impact on people’s quality of life...oftentimes where I’m seeing them is they’re not able to do certain things, they’ve stopped doing things that they enjoy because of the physical and mental health issues or they’re finding things much harder to do than what they once did. And to be able to impact on that and give them a little bit of that back it adds to the quality of life and that’s meaningful to me.”*

(Exercise Specialist 7).

Home care providers such as personal support workers identified the important impact of their care involvement on older adults’ comfort level and safety in staying in their own homes. They felt satisfied in being able to help clients reach their goals and maintain their independence in their own home.

*“The rewards are watching the improvements, if there’s any improvement at all in their condition. Restoring their confidence and dignity and their abilities to do as much for themselves as they can and stay in their own home. It’s hard to put into words.”*

(Personal Support Worker 16).

They described the value of home visits that provided an important opportunity for older adults to socialize as well as receive care.

*“I guess mostly comfort; making them feel comfortable in their own home; making them happy to see you; getting rid of uneasiness, because a lot of times especially at that age where less and less people are coming to visit them and what not; socialization; just improving their state of mind and physical comfort.”*

(Personal Support Worker 10).

Healthcare providers described the rewards of feeling appreciated by older adults with MCC. They felt that older adults valued their suggestions and expertise in managing complex chronic conditions. Listening to and talking with older adults with MCC and their family caregivers about their concerns elicited feelings of gratitude especially when clients and families felt stressed.

*“Getting their appreciation is always rewarding. Sometimes they even will appreciate as much as you calling to check in and see how things are going. For example, if they’ve recently started insulin, they enjoy that follow-up phone call and also the opportunity to ask questions.”*

(Nurse 4).

Providers explained that older adults with MCC sometimes felt neglected by the healthcare system which led them to appreciate the care and attention they received from providers.

*“Sometimes they feel like they’re kind of getting ignored by the system a bit, I think. Like, they’re sort of getting left in the dust or they’re not as important anymore because they’re older and so, they’re very appreciative; they can be very appreciative. The family can be very appreciative, too.”*

(Physician 18).

#### Recommending ways to address the challenges of the healthcare system

Healthcare providers made recommendations regarding how to improve care for community-living older adults with MCC and their caregivers. These recommendations were related to improving the organization of care and service delivery for older adults with MCC, providing more time for older adults with MCC to discuss their health conditions, and ensuring that older adults with MCC have access to home care supports.

***Improving care coordination: “a more streamlined healthcare system.”*** Healthcare providers recommended restructuring of the healthcare system to improve care coordination between providers and sectors and improving efficiencies in care.

*“Definitely a more streamlined healthcare system. Like there’s a lot of hoops to jump through and papers to fill out and this person has to refer to this. Like it’s just not very easy for people to navigate. It’s just not an easy system and there’s a lot of wasted time with repeat assessments or two people doing the same job. It’s like a process that just makes the healthcare system not really time effective and cost effective. It’s a lot of wasted time.”*

(Home Care Case Manager 19).

To improve coordination between primary care, community care and acute care services, providers recommended that all providers have access to the same electronic medical record to obtain detailed and comprehensive information about older adults with MCC: “*the shared patient record is key*” (Dietitian 6). Providers acknowledged that older adults with MCC often transitioned from community to acute care and back to community and indicated the need for a “*stronger connection between the hospital and the community agency*” (Social Worker 1). They discussed how home care services were at times delayed when clients with MCC were discharged from hospital and putting in home care services needed to be done more quickly.

***Improving primary care: “longer more regular visits.”*** Healthcare providers recommended that older adults with MCC receive more regular and longer primary care visits to monitor the progress of conditions and improve care management. They noted that older adults with MCC have many concerns that they would like to discuss with their providers and a single visit, often limited to addressing a single concern, was insufficient to address their complex needs.

*“The elder person or even the young senior, when they’ve got a whole bunch of things going on and they all meld together as they do, when they see the doctor or the nurse practitioner, even frequently once a month, they have a million things to tell them and for the doctor or the nurse practitioner, it’s overwhelming. And what we have certainly found works best is seeing.the elder once a week routinely, and addressing in small bits, their needs.”*

(Social Worker. 30)

Providers also suggested longer primary care clinic visits to fully address client questions and better manage MCC. Effective communication between clients and providers was seen as necessary for quality of care.

*“There’s so many medical conditions to look at and the visits, in order to service them properly I always feel the visits need to be much longer than is allotted to answer questions with very long answers to questions or lack of understanding of their diseases.”*

(Nurse 20).

***Increasing home care supports: “they need more home care.”*** Healthcare providers identified the need for more home care supports for older adults with MCC and their caregivers to improve their ability to age at home. More than half of providers working in home care reported that clients require more home care supports compared to less than half of providers in primary care. *“[Home care] is budget limited…so not only am I limited in a visit but I am time limited with respect to number of treatments and calendar time that I can treat these people*” (Physiotherapist 7). They indicated that the amount of home care should be increased including respite to support caregivers. Providers suggested that continuity of care providers (e.g., personal support workers) be improved so there could be improved understanding of and response to the complex conditions of older adults. Home care providers spoke of the human resource issues and the need for more people in the home care workforce to address the need of this group of older adults.

*“It just seems we are always short-staffed, the caseloads sometimes are really high numbers. It’s hard to accommodate everybody’s needs in terms of agency services. I think we just need more people in the workforce with this aging population.”*

(Home Care Case Manager 20).

## Discussion

This study aimed to explore the experiences of a broad group of community-based primary and home care providers in supporting older adults living in the community to manage MCC. Study findings provide important new understandings of the experiences of this group of providers (e.g., nurses, personal support workers, social workers, physiotherapists, and others) in caring for these individuals. First, this study found that providers used a number of key strategies to manage the complexity associated with MCC. Recognizing that older adults with MCC often have many prescription medications with a high risk of medication interactions and side effects, these providers sought to optimize medications use by completing regular medication reviews, simplifying dosing of medications, deprescribing and finding a balance between under and over treating with medications. Previous literature has described primary care provider roles such as adjusting medication regimens, deprescribing, and tailored, patient-centered approaches to optimize medication use [19, 35, 36]..

Study findings provide new insights into how these healthcare providers recognized the need to holistically address the interconnections not only among multiple chronic conditions but also social conditions in the lives of older adults with MCC. They implemented strategies to reduce out-of-pocket costs related to medications, supplies, transportation, and support services that would assist older adults to better manage their MCC. Two recent reviews have found that the social determinants of health have not been well integrated into conceptualizations of multimorbidity [37, 38]. A scoping review by McGilton et al. (2018) found that social determinants of health such as income, access to resources, and social support networks are largely absent in current conceptualizations of multimorbidity and yet critically influence the health and social care needs of older adults with MCC [37]. On the contrary, our study findings showed that community-based healthcare providers do consider and address these issues in managing care for older adults with MCC.

Second, study findings indicate that participants used a number of person-centred care approaches such as individualizing care to the unique needs of each person based on their preferences and goals. Care decisions were made using a person-centred approach and focused on optimizing quality of life and enhancing daily function to encourage independence for older adults with MCC. These findings are similar to another study exploring primary care in the Netherlands that reported that general practitioners used an individualized approach in managing MCC, implemented shared decision-making with clients, and ensured that client-centredness emerged at the forefront of all their decisions [18]. Interestingly, these findings are inconsistent with the views of older adults with MCC and their caregivers who feel they are seldom involved as active participants in care decisions [14, 15]. The current study is unique as it provides a Canadian lens on how home care and primary care providers other than family physicians implement person-centred care for older adults with MCC in the community. The inclusion of personal support workers in the current study provides an important understanding of the experiences of unregulated workers in caring for persons with MCC.

Third, study results indicate that these healthcare providers recognized the critical roles played by family and friend caregivers to support older adults with MCC living in the community and included them in their care approaches. Providers offered emotional support and education to help increase caregiver self-efficacy in helping their family members. Providers in the current study also advocated for peer support and respite for caregivers. Previous literature on primary care providers who care for older adults with MCC has acknowledged the importance of caregivers but has focused primarily on family physicians [13, 17]. Clearly, there is a need to ensure that those who are supporting older adults with MCC are provided with resources to maintain their health and help them feel confident in caring for their family member.

Fourth, study results highlight the unique contribution of providers from a wide array of professions and settings in understanding the challenges faced by older adults with MCC and in working together to address these challenges. Healthcare providers such as nurses and social workers felt that older adults were more willing to share some pieces of information important to managing their conditions with them, rather than physicians as suggested in the quote: “they don’t want to tell the doctor that.” This suggests the value of an interprofessional team, with unique perspectives and skills, contributing to health care decision making to support older adults with MCC.

Participants described the challenges of poor team communication that negatively impacted their care provision. This study highlighted the challenges that home care providers experienced in lacking access to a common medical record and timely information sharing from other team members. Previous research with staff in home care settings who cared for older persons with MCC describes somewhat similar challenges in relation to uncertainty of team member responsibilities, and lack of trust between team members [24].

Next, primary care and home care providers described both challenges and rewards in caring for older adults with MCC. Challenges discussed included finding time to address multiple and complex needs, respecting client healthcare choices that they did not agree with, providing support to stressed caregivers, and linking clients to needed resources and services in the face of financial constraints. Our findings are consistent with challenges reported in other literature [18, 20, 22]. Although most previous literature is focused on the challenges of supporting care for older adults with MCC, our study uniquely reveals the personal rewards that healthcare providers experienced in caring for this group. Providers felt rewarded by seeing improvements in older adults’ health, functioning and quality of life, and being appreciated by them and their caregivers. One study of community care providers also explored the rewards in caring for older adults with MCC, but this was from the perspective of senior’s mental health case managers [39]. Perrella et al. (2018) found that case managers felt rewarded by: (a) facing complexity in caring for older adults with MCC, (b) enhancing their skills and knowledge, and (c) gaining human connections with their clients and colleagues [39]. Our study adds to the understanding of rewards experienced by a broad range of community-based providers caring for older persons with MCC.

Finally, our study contributes an understanding of the recommendations primary and home care providers have to improve care for older adults with MCC based on their extensive clinical experiences. Their recommendations address some components of an integrated system of healthcare such as coordination of person-focused care; the integration of biomedical, psychological and social dimensions of health; and information systems structured around service delivery [40]. Some models have been implemented to promote such comprehensive interprofessional primary care delivery for older adults with MCC such as the Geriatric Resources for Assessment and Care of Elders (GRACE) model, Guided Care and the IMPACT (Interprofessional Model of Practice for Aging and Complex Treatments) clinic [41–43]. These models have had a positive impact on outcomes such as increased interprofessional collaboration and decreased emergency visits [41–43].

A unique finding was that healthcare providers in the current study, particularly home care providers, recommended having a common electronic medical record for older adults with MCC that all providers can access in order to improve communication and coordination between services such as primary care, home care, and acute care services. Persons with MCC have been found to be overburdened in managing personal health information and technological applications such as electronic medical records can be an effective structural solution for information exchange between providers and clients [44].

Half of the study participants were nurses including Registered Nurses, Registered Practical Nurses, a Nurse Practitioner, and a Nurse Case Manager. These primary care and home care providers played key roles in supporting older adults with MCC and their caregivers. It has been argued that the increasing prevalence of chronic diseases and the shortage of primary care physician are powerful forces driving primary care towards more Nurse Practitioner and Registered Nurse participation in provision of chronic care services [45]. Further research is needed to explore how these professionals can shape community care for the growing population of older adults with MCC.

### Strengths and limitations

Study strengths included: (a) the use of ID design that facilitated the examination of the complexity of this topic; (b) a large sample of healthcare providers from diverse disciplines who worked in primary and home care settings; (c) representation of providers from two large Canadian provinces; and (d) a rigorous data analytic approach to promote study credibility including regular meetings of a research team members with research expertise in qualitative research, community health, older adults with MCC, and family caregivers. There were a number of study limitations. First, participants included only small numbers of certain professional groups such as home care case managers, nurse practitioners, pharmacists and dietitians. These healthcare providers play key roles in supporting older adults with MCC and could provide more insight related to their care of this group. As with all qualitative research, any claims of generalizability are tenuous, and transferability is suited best to similar samples and settings.

## Conclusions

The experiences of these primary care and home care providers reveal that they use a broad array of relevant strategies and approaches to support the complex care of older adults with MCC living in the community. They took into consideration the complex interweave of chronic conditions with psychological, social and financial issues in the lives of these individuals as well as the important role of family and friend caregivers. However, healthcare providers also identified many challenges they faced in providing care to this vulnerable group, not only at the level of patient-provider interactions, but also at the levels of interprofessional teams and healthcare systems. They identified the need for a more comprehensive and coordinated approach to better address the needs of these older adults. This study supports the need for an integrated model of care in the community to enable this population to continue to age at home with the best possible quality of life.

## Data Availability

The data for this research consist of interview transcripts. Raw data cannot be publicly released due to the risk of compromising participant confidentiality.

## Abbreviation

MCC: Multiple chronic conditions

## References

[1] Boyd CM, Fortin M (2010). Future of multimorbidity research: how should understanding of multimorbidity inform health system design?. Public Health Rev.

[2] Fried TR, O'leary J, Towle V, Goldstein MK, Trentalange M, Martin DK (2014). Health outcomes associated with polypharmacy in community-dwelling older adults: a systematic review. J Am Geriatr Soc.

[3] World Health Organization (WHO). World report on aging and health. 2015. http://www.who.int/ageing/events/world-report-2015-launch/en/. Accessed 27 Dec 2018.

[4] Salive ME (2013). Multimorbidity in older adults. Epidemiol Rev.

[5] Fortin M, Stewart M, Poitras M, Aimirall J, Maddocks H (2012). A systematic review of prevalence studies on multimorbidity: toward a more uniform methodology. Ann Fam Med.

[6] Marengoni A, Angleman S, Melis R, Mangialasche F, Karp A, Garmen A (2011). Aging with multimorbidity: a systematic review of the literature. Ageing Res Rev.

[7] Prince MJ, Wu F, Guo Y, Robledo LMG, O'Donnell M, Sullivan R (2015). The burden of disease in older people and implications for health policy and practice. Lancet..

[8] Griffith LE, Gruneir A, Fisher K, Panjwani D, Gandhi S, Sheng L (2016). Patterns of health service use in community living older adults with dementia and comorbid conditions: a population-based retrospective cohort study in Ontario. Canada BMC Geriatr.

[9] Fisher K, Griffith L, Gruneir A, Panjwani D, Gandhi S, Sheng L, Gafni A, Patterson C, Markle-Reid M, Ploeg J (2016). Comorbidity and its relationship with health service use and cost in community-living older adults with diabetes: a population-based study in Ontario. Canada Diabetes Res Clin Pract.

[10] Gruneir A, Griffith L, Fisher K, Panjwani D, Gandhi S, Sheng L, Patterson C, Gafni A, Ploeg J, Markle-Reid M (2016). (2016). Increasing comorbidity and health services utilization in older adults with prior stroke. Neurology..

[11] Uhlig K, Leff B, Kent D, Dy S, Brunnhuber K, Burgers JS (2014). A framework for crafting clinical practice guidelines that are relevant to the care and management of people with multimorbidity. J Gen Int Med.

[12] Bähler C, Huber CA, Brüngger B, Reich O. Multimorbidity, health care utilization and costs in an elderly community-dwelling population: a claims data based observational study. BMC Health Serv Res. 2015:15–23. 10.1186/s12913-015-0698-2 PMC4307623 25609174

[13] Gill A, Kuluski K, Jaakkimainen L, Naganathan G, Upshur R, Wodchis WP (2014). "where do we go from here?" health system frustrations expressed by patients with multimorbidity, their caregivers and family physicians. Healthc Policy.

[14] Ploeg J, Matthew-Maich N, Fraser K, Dufour S, McAiney C, Kaasalainen S (2017). Managing multiple chronic conditions in the community: a Canadian qualitative study of the experiences of older adults, family caregivers and healthcare providers. BMC Geriatr.

[15] Ploeg J, Canesi M, Fraser KD, McAiney C, Kaasalainen S, Markle-Reid M (2019). Experiences of community-dwelling older adults living with multiple chronic conditions: a qualitative study. BMJ Open.

[16] Junius-Walker U, Wrede J, Schleef T, Diederichs-Egidi H, Wiese B, Hummers-Pradier E (2012). What is important, what needs treating? How GPs perceive older pateints’ multiple health problems: a mixed methods research study. BMC Res Notes.

[17] Kuluski K, Gill A, Naganathan G, Upshur R, Jaakkimainen RL, Wodchis WP (2013). A qualitative descriptive study on the alignment of care goals between older persons with multi-morbidities, their family physicians and informal caregivers. BMC Family Pract.

[18] Luijks HD, Loeffen MJ, Lagro-Janssen AL, Van Weel C, Lucassen PL, Schermer TR (2012). GPs’ considerations in multimorbidity management: a qualitative study. Br J Gen Pract.

[19] Schuling J, Gebben H, Veehof LJ, Haaijer-Ruskamp FM (2012). Deprescribing medication in very elderly patients with multimorbidity: the view of Dutch GPs. A qualitative study. BMC Fam Pract.

[20] Sinnott C, McHugh S, Browne J, Bradley C (2013). GPs’ perspectives on the management of patients with multimorbidity: systematic review and synthesis of qualitative research. BMJ Open.

[21] Bower P, Macdonald W, Harkness E, Gask L, Kendrick T, Valderas JM (2011). Multimorbidity, service organization and clinical decision making in primary care: a qualitative study. J Fam Pract.

[22] Ferris R, Blaum C, Kiwak E, Austin J, Esterson J, Harkless G (2018). Perspectives of patients, clinicians, and health system leaders on changes needed to improve the health care and outcomes of older adults with multiple chronic conditions. J Aging Health.

[23] Fried TR, Tinetti ME, Iannone L (2011). Primary care clinicians' experiences with treatment decision making for older persons with multiple conditions. Arch Intern Med.

[24] Larsen A, Broberger E, Petersson P (2017). Complex caring needs without simple solutions: the experience of interprofessional collaboration among staff caring for older persons with multimorbidity at home care settings. Scand J Caring Sci.

[25] Thorne S (2016). Interpretive description: qualitative research for applied practice.

[26] Patton MQ (2015). Qualitative research & evaluation methods.

[27] World Health Organization (WHO). Cardiovascular diseases (CVDs). 2017. https://www.who.int/en/news-room/fact-sheets/detail/cardiovascular-diseases-(cvds). Accessed 4 April 2019.

[28] Government of Alberta. Population statistics. 2018. https://www.alberta.ca/population-statistics.aspx Accessed 4 April 2019.

[29] Statistics Canada. The Canadian population in 2011: Age and sex. Statistics Canada 2012. https://www12.statcan.gc.ca/census-recensement/2011/as-sa/98-311-x/98-311-x2011001-eng.cfm. .

[30] Braun V, Clarke V (2006). Using thematic analysis in psychology. Qual Res in Psychol.

[31] NVivo qualitative data analysis Software; QSR International Pty Ltd. Version 10, 2012.

[32] Lincoln YS, Guba EG (1985). Naturalistic inquiry.

[33] Creswell JW (2013). Qualitative inquiry and research design: choosing among five approaches.

[34] Canadian Institutes of Health Research, Natural Sciences and Engineering Research Council of Canada, and Social Sciences and Humanities Research Council of Canada. Tri-Council Policy Statement: Ethical Conduct for Research Involving Humans. 2014. http://www.pre.ethics.gc.ca/pdf/eng/tcps2-2014/TCPS_2_FINAL_Web.pdf. Accessed 4 April 2019.

[35] McMullen CK, Safford MM, Bosworth HB, Phansalkar S, Leong A, Fagan MB (2015). Patient-centered priorities for improving medication management and adherence. Patient Educ Couns.

[36] Steinman MA, Low M, Balicer RD, Shadmi E (2018). Impact of a nurse-based intervention on medication outcomes in vulnerable older adults. BMC Geriatr.

[37] McGilton KS, Vellani S, Yeung L, Chishtie J, Commisso E, Ploeg J, Andrew MK, Ayala AP, Gray M, Morgan D, Chow AF (2018). Identifying and understanding the health and social care needs of older adults with multiple chronic conditions and their caregivers: a scoping review. BMC Geriatr.

[38] Northwood M, Ploeg J, Markle-Reid M, Sherifali D (2018). Integrative review of the social determinants of health in older adults with multimorbidity. J Adv Nurs.

[39] Perrella A, McAiney C, Ploeg J (2018). Rewards and challenges in caring for older adults with multiple chronic conditions: perspectives of seniors’ mental health case managers. Can J Commun Ment Health.

[40] Valentijn PP, Schepman SM, Opheij W, Bruijnzeels MA (2013). Understanding integrated care: a comprehensive conceptual framework based on the integrative functions of primary care. Int J Integr Care.

[41] Counsell SR, Callahan CM, Clark DO, Tu W, Buttar AB, Stump TE, Ricketts GD (2007). Geriatric care management for low-income seniors: a randomized controlled trial. JAMA..

[42] Boyd CM, Reider L, Frey K, Scharfstein D, Leff B, Wolff J, Groves C, Karm L (2010). The effects of guided care on the perceived quality of health care for multi-morbid older persons: 18 month outcomes from a cluster-randomized controlled trial. J Gen Intern Med.

[43] Tracy CS, Bell SH, Nickell LA (2013). The IMPACT clinic: innovative model of interprofessional primary care for elderly patients with complex healthcare needs. Can Fam Physician.

[44] Ancker JS, Witteman HO, Hafeez B, Provencher T, Van de Graaf M, Wei E (2015). The invisible work of personal health information management among people with multiple chronic conditions: qualitative interview study among patients and providers. J Med Internet Res.

[45] Bodenheimer T, Bauer L (2016). Rethinking the primary care workforce – an expanded role for nurses. NEJM..

